# Validating the use of the revised childbirth experience questionnaire in Hong Kong

**DOI:** 10.1186/s12884-022-04456-x

**Published:** 2022-02-15

**Authors:** Kris Y. W. Lok, Heidi S. L. Fan, Rachel W. T. Ko, Jojo Y. Y. Kwok, Janet Y. H. Wong, Daniel Y. T. Fong, Noel W. M. Shek, Hextan Y. S. Ngan, Edmond P. H. Choi

**Affiliations:** 1 School of Nursing, Li Ka Shing Faculty of Medicine, The University of Hong Kong, Pok Fu Lam, Hong Kong; 2 Department of Obstetrics and Gynaecology, Queen Mary Hospital, University of Hong Kong, Pok Fu Lam, Hong Kong

## Abstract

**Objective:**

To evaluate the psychometric properties of the traditional Chinese version of the Childbirth Experience Questionnaire (CEQ 2.0) and assess the childbirth experiences of Chinese women.

**Methods:**

A cross-sectional survey was conducted in Hong Kong from July 2020 to February 2021. In total, 975 mothers, who could read traditional Chinese and gave birth in 2020 or 2021, were included in the analysis. Data were fitted into the model proposed by the original developers using the confirmatory factor analysis. The data were then randomly split into training and validation sets for exploratory and confirmatory factor analyses. Childbirth experiences were assessed. Factor structure, internal construct validity, internal consistency, and known-group validity were assessed.

**Results:**

The originally proposed CEQ2.0 model showed a poor fit. An exploratory factor analysis identified a revised four-factor model (CEQ2.0-R) on a randomly split sample, which showed a satisfactory fit (CFI=0.912; TLI=0.884; SRMR=.053; RMSEA=0.072) on the other split sample. The revised scale comprised 13 items and four domains: (1)*“Own capacity”* (6 items), (2) *“General support”* (3 items), (3) *“Perceived safety”* (2 items), *and* (4) *“Professional support”* (2 items)*.* CEQ2.0-R showed high internal construct validity and reliability. It can differentiate between participants with different characteristics, including parity, oxytocin augmentation, and companionship during labour. The childbirth experiences of the participants were merely positive, and participants reported that more support from midwives is needed.

**Conclusions:**

CEQ2.0-R can adequately describe the childbirth experiences of women in Hong Kong. The questionnaire is easy to be administer and can be used to assess several domains of the childbirth experiences. It may be useful to evaluate the aspects of support needed during childbirth.

**Supplementary Information:**

The online version contains supplementary material available at 10.1186/s12884-022-04456-x.

## Background

Measuring childbirth experiences is an important tool to assist clinicians and researchers in quantifying women’s experiences relating to labour and birth to evaluate practice. With the current pandemic and government restrictions on non-accompanied labour [1], it is important to measure the impact of these factors on outcomes of women’s labour experiences. The lack of a robust, validated tool for evaluating labour experiences in Hong Kong during the pandemic is a topical issue. Even though various tools measuring women’s perinatal experiences exist [2], in the literature, most scales lack of complete testing of the psychometric properties [2].

In 2010, a multidimensional Childbirth Experience Questionnaire (CEQ) [3] was developed in Sweden, measuring four domains of the childbirth experiences. The four domains include: *Own capacity*, *Perceived safety*, *Professional support*, and *Participation*. The instrument was robust and reliable and has been translated and validated in several languages [4–7] and used in studies of culturally diverse samples [8–10]. However, CEQ was recently revised in Sweden by the same team of developers, as it was found that two domains, *Participation* and *Professional support*, showed weaker performance. A total of 14 new items were revised. In *Professional support*, reversed items were developed to avoid high ceiling effects. In *Participation*, more relevant items relating to information and decision-making were added [11]. The Childbirth Experience Questionnaire (CEQ2.0) was then developed. It was validated and showed good criterion validity in relation to the nationally used Maternity Survey, test-retest reliability, and differences between known groups, in both primiparous and multiparous women [11].

Measuring the impact of an intervention or a policy on women’s childbirth experiences is as important as measuring its impact on outcomes such as mode of birth, perinatal outcomes, maternal postpartum health, which is scarce in the literature. As aforementioned, there was a lack of a reliable instrument that measures the birthing experiences in Chinese. A recent study has validated CEQ in the Chinese population [6]. As CEQ was modified and CEQ2.0 was developed, the objectives of this study are to validate CEQ2.0 in the Chinese population and to assess its effectiveness in evaluating labour experiences.

## Methods

### Design, setting, and participants

A cross-sectional survey was conducted in Hong Kong from July 2020 to February 2021. The details of the study were published elsewhere [12]. In brief, participants were recruited by (1) a research assistant who distributed leaflets to women in the obstetrics clinic, (2) an independent researcher in five maternal and child health centers in three regions in Hong Kong, and (3) online promotions on social media and mothers’ groups. The participants completed the survey online or were interviewed by the independent researcher in the maternal and child health centers. The survey, available in English and Traditional Chinese, includes sociodemographic characteristics, obstetrics, maternal health histories, infant feeding practices, the fear level, depressive symptoms, and the childbirth experiences. Eligible participants included women (1) aged 18 or above who (2) were pregnant or gave birth since 2020. As the objective of this study was to validate the Chinese version of CEQ2.0, only women who gave birth and completed the traditional Chinese CEQ2.0 were included in the analysis.

### Childbirth Experience Questionnaire (CEQ2.0)

The childbirth experiences of the women were measured using CEQ2.0. We obtained permission to use the questionnaire from Dr. A. Dencker, who was the developer of the original Swedish version of CEQ2.0 [11]. In this study CEQ2.0 was first translated from English to traditional Chinese using a forward-backward translation procedure. The English CEQ2.0 was forward translated into Chinese by two bilingual translators who are native Chinese speakers and health professionals. Then, both translators compared their translations in a consensus meeting with a senior team member and discussed any discrepancies. Two other native Chinese-speaking bilingual translators who were completely blinded to CEQ2.0 translated the Chinese version back into English and this translation was compared with the English version by a native English speaker (to identify discrepancies in the Chinese translation). The final Chinese version of CEQ2.0 was then prepared.

CEQ2.0 comprises 22 statements assessing four domains of the childbirth experiences. These include (i) *Own capacity*, (ii) *Perceived safety*, (iii) *Professional support*, and (iv) *Participation*. Responses are scored using a 4-point Likert scale ranging from 4 (totally agree), 3 (mostly agree), 2 (mostly disagree), to 1 (totally disagree). Three items referring to labour pain, sense of security, and control are assessed with visual analogue scales (VAS). The VAS-scale scores are transformed to categorical values, 0-40=1, 41-60=2, 61-80=3, and 81-100=4. Item rating was summed and a higher score indicates a better childbirth experience.

### Sample size

The planned sample size was 220 women. This was based on the recommendation of a sample size of ten times the number of observed variables in the health measurement tool being evaluated [13]. CEQ2.0 comprises 22 items; therefore 220 completed questionnaires would be required. Assuming a 70% response rate from the original Swedish study and a 20% attrition rate, approximately 400 women are needed to achieve a final sample size. However, as this study is not the primary study outcome of the survey, we calculated the sample size of the survey based on the main study outcome. Therefore, we obtained a larger sample size.

### Statistics and data analysis

Baseline descriptive statistics were reported in all participants who completed a baseline CEQ2.0, using frequencies with proportions or mean values with standard deviation (SD). In addition, the mean and SD of each item of CEQ2.0 were presented. To validate CEQ2.0 in the Chinese population, the factor structure, internal validity, known-group validity, and reliability of the scale were evaluated.

### Factor structure

First of all, data were fitted into the model proposed by the original authors using the confirmatory factor analysis (CFA) [11]. However, the model did not fit well with the data. Therefore, the data were randomly split into a training set (*n*=487) and a validation set (*n*=488).

The training set was used in the exploratory factor analysis (EFA) using the maximum likelihood factoring with promax rotation of factors. The number of factors was identified by using scree plot. The Bartlett’s test of sphericity was used to test the assumption of sphericity, and the Kaiser-Meyer-Olkin (KMO) test was used to test the sampling adequacy. Items with a factor loading ≥0.4 were assigned to the factor [14], and a new factor structure (CEQ2.0-R) was developed.

Using the validation set, we conducted a confirmatory factor analysis (CFA) to assess the factor structure suggested in the EFA. Factor structure was tested in the models using model fit statistics; χ2, the comparative fit index (CFI) [15], Tucker–Lewis index (TLI), standardized root mean square residual (SRMR), and root mean square error of approximation (RMSEA). A CFA model was considered as a relatively good fit if the CFI and TLI values are close to 0.95, the SRMR value is close to 0.08, and the RMSEA value is close to 0.06 [16]. Hu and Bentler suggested using the word “close to” because the fit indices did not work well equally under different conditions [16].

### Internal construct validity

Item-total correlation was used to assess the internal construct validity in the whole sample. A correlation coefficient should be ≥0.2 to be remained in the scale [17].

### Internal consistency

To assess internal consistency, Cronbach’s alpha coefficient (α) was calculated for each of the four factors in the whole sample, using the same item loadings as the CFA for CEQ2.0, α ≥0.70 to 0.90, was considered to have good internal consistency [18].

### Known-group validity

We used a known-group validity assessment to test the ability of CEQ2.0-R to differentiate the groups. Independent t-tests were used to test the differences in the four factors and the total scores between parity, oxytocin augmentation, and mode of birth based on the previous studies [11, 19]. In addition, the use of pain medication and companionship during labour were also examined as studies show that there were differences in childbirth experiences between groups [20, 21]. Cohen’s d effect sizes *(d)* were calculated and considered as trivial (<0.2), small (≥0.2 and <0.5), moderate (≥0.5 and <0.8) or large (>0.8) [22].

Statistical analyses were conducted using Stata Statistical Software: Release 16 (StataCorp. 2013. College Station, TX: StataCorp LP). A .05 level of significance was used throughout the study.

## Results

Overall, 1035 participants who were in the postpartum period completed the Chinese version of the survey. We excluded 60 participants because they had fixed or random response for CEQ2.0. Therefore, 975 participants were included in the analysis. The median time of completing the questionnaire is 4.1 months postpartum. The majority of participants were born in Hong Kong (88.5%), were in employment (66.7%), were married (97.1%), primiparous (71.1%), had a spontaneous vaginal birth (67.9%), and had their birth in public hospitals (74.5%) (Table 1).

**Table 1:** Descriptive characteristics of the study population

Variables	Sample (***n***=975)
Maternal age, years, mean (SD)	32.5 (4.1)
Gestational age weeks, mean (SD)	38.5 (1.6)
Place of birth, n (%)	
Hong Kong	863 (88.5)
Other	112 (11.5)
Education, n (%)	
Secondary school or below	395 (40.5)
College or above	580 (59.5)
Current employment status, n (%)	
Full-time or part-time employment	650 (66.7)
Unemployed	325 (33.3)
Marital status, n (%)	
Married	947 (97.1)
Not married but live with partner	26 (2.7)
Separated/ divorced	2 (0.2)
Monthly family income (HKD), n (%)	
<$15,000	28 (2.9)
$15,000-$24,999	121 (12.4)
$25,000-$34,999	208 (21.3)
$35,000-$44,999	222 (22.8)
≥$45,000	396 (40.6)
Parity	
Primiparous	693 (71.1)
Multiparous	282 (28.9)
Length of stay in antenatal ward, n (%)	
Admitted to labour ward directly	142 (14.6)
<1 hour	54 (5.5)
1-5 hours	235 (24.1)
6-11 hours	235 (24.1)
12-23 hours	193 (19.8)
≥24 hours	116 (11.9)
Mode of birth, n (%)	
Spontaneous vaginal birth	662 (67.9)
Assisted vaginal birth	51 (5.2)
Planned caesarean	162 (16.6)
Emergency caesarean	100 (10.3)
Required oxytocin augmentation, n (%)	
Oxytocin augmentation	408 (41.9)
No oxytocin augmentation	567 (58.2)
Companionship during labour	
Yes	623 (63.9)
No	352 (36.1)
Place of birth, n (%)	
Private hospital	249 (25.5)
Public hospital	726 (74.5)

*SD* standard deviation

### Factor analysis

A CFA was conducted on the whole sample, and a poor fit of the data into the model proposed by the original authors [11] (CFI=0.656; TLI=0.608; SRMR=0.102; RMSEA=0.104) (see Table 2). The factor structure of CEQ2.0 is shown in Appendix I. Bartlett’s test of sphericity was significant (*P*<.001), and the KMO measure of sampling adequacy was 0.851, which supports the use of factor analysis for this data. Therefore, the data were randomly split into a training set for conducting the EFA (*n*=487) and a validation set for conducting the CFA (*n*=488).

**Table 2:** Goodness of fit of the indicators in the original CEQ2 factor model and CEQ2-R factor model

	Chi-square value of model fit	*DF*	*P* value	CFI	TLI	SRMR	RMSEA	90%CI
Model by the original authors	2348.143	203	<.001	0.656	0.608	0.102	0.104	0.100-0.108
Proposed model	209.406	59	<.001	0.912	0.884	0.053	0.072	0.062-0.083

*DF* degree of freedom, *AIC* Akaike’s Information Criterion, *BIC* Bayesian Information criterion, *CFI* comparative fit index, *TLI* Tucker-Lewis index, *SRMR* standardized root mean square residual, *RMSEA* root mean square error of approximation, *CI* confidence interval

**Fig. 1:**
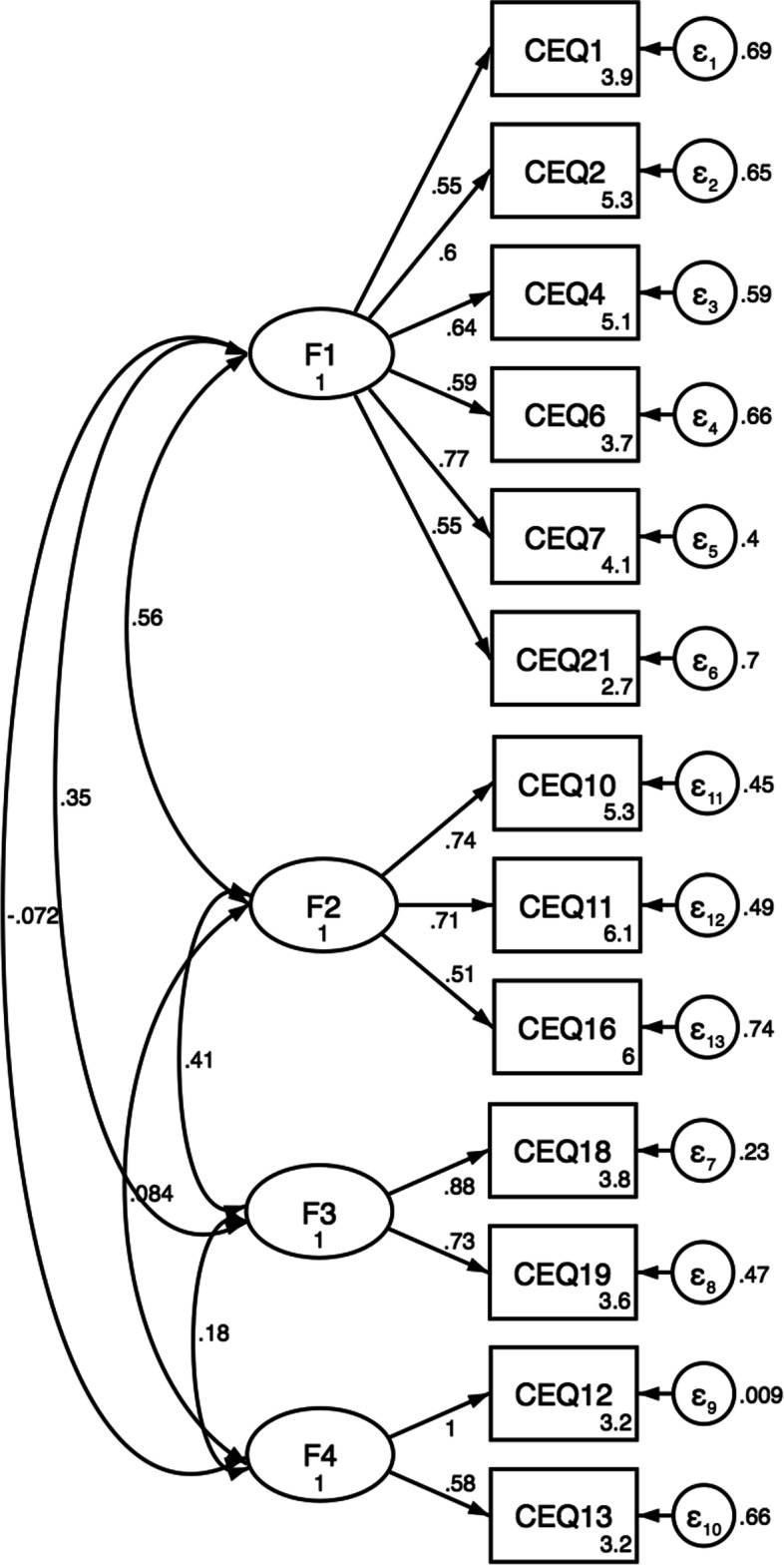
Factor structure of CEQ2.0-R

The scree plot suggested a four-factor structure, and the corresponding rotated factor loadings showed the factors were *“Own capacity”, “General support”, “Perceived safety”* and *“Professional support”* (Table 3) . The four factors accounted for 59.8%, 22.7%, 9.64%, and 7.8% of the variances, respectively. Nine items (item 3, 5, 8, 9, 14, 15, 17, 20 and 22) had factor loadings <0.4 and were removed. The resulting 13 items formed CEQ2.0-R. The factors consist of 2 to 5 items, i.e. “*Own capacity*” (6 items), “*General support*” (3 items), “*Perceived safety*” (2 items), and “*Professional support*” (2 items). The factor structure of CEQ2.0-R is shown in Fig. 1. CEQ2.0-R improved the fit indices (CFI=0.912; TLI=0.884; SRMR=0.053; RMSEA=0.072) when compared with the original model (Table 2).

**Table 3:** Exploratory factor analysis (EFA) results

Own capacity		
1	Labour and birth went as I had expected	0.6122
2	I felt strong during labour and birth	0.5175
4	I felt capable during labour and birth	0.5726
5^ab^	I was tired during labour and birth	0.0849
6	I felt happy during labour and birth	0.8089
7	I felt that I handled the situation well	0.8918
20^abc^	As a whole, how painful did you feel childbirth was?	-0.021
21^c^	As a whole, how much control did you feel you had during childbirth?	0.4189
Variance		59.8%
General support		
10	Both my partner and I were treated with warmth and respect	0.7738
11	Received the information I needed during labour and birth	0.8082
16	My impression of the team’s medical skills made me feel secure	0.5131
8^ab^	I wish the staff had listened to me more during labour and birth	0.0194
9^b^	I took part in decisions regarding my care and treatment as much as I wanted	0.3498
Variance		22.7%
Perceived safety		
3^ab^	I felt scared during labour and birth	0.3575
17^b^	I have many positive memories from childbirth	0.3864
18^a^	I have many negative memories from childbirth	0.8586
19^a^	Some of my memories from childbirth make me feel depressed	0.7857
22^bc^	As a whole, how secure did you feel during childbirth?	0.2785
Variance		9.64%
Professional support		
12^a^	I would have preferred the midwife to be more present during labour and birth	0.6830
13^a^	I would have preferred more encouragement from the midwife	0.8478
14^b^	The midwife conveyed an atmosphere of calm	-0.2642
15^b^	The midwife helped me to find my inner strength	-0.2342
Variance		7.8%

Exploratory factor analysis (EFA) using maximum likelihood factoring with Promax rotation of factors. Point of inflexion of the curve was used to determine the number of factors used in the analysis

^a^Items are reverse record. The score of all reversed items were reordered in the EFA so that all items measured in the same direction

^b^Items were removed because the factor loading was <0.4

^c^The items were assessed with visual analogue scales. The items were transformed into a 4-point Likert scale

### Internal construct validity and reliability

We used the item-total correlation to assess the internal construct validity. Satisfactory item-total correlation was shown in all the items (see Table 4). In addition, good reliability was shown in the four domains. The α was ≥0.7 in all the domains.

**Table 4:** Descriptive statistics, internal construct validity and reliability (*n*=975)

		Item total correlation values^a^	Mean score (SD)^a^	Response distribution of individual items^b^ N (%)			
				Totally disagree	Mostly disagree	Mostly agree	Totally agree
Domain: Own capacity			2.85 (0.50)				
1	Labour and birth went as I had expected	0.61	2.76 (0.70)	57 (5.9)	216 (22.2)	609 (62.5)	93 (9.5)
2	I felt strong during labour and birth	0.58	3.11 (0.61)	15 (1.5)	86 (8.8)	649 (66.6)	225 (23.1)
4	I felt capable during labour and birth	0.59	3.09 (0.61)	11 (1.1)	106 (10.9)	643 (66.0)	215 (22.1)
6	I felt happy during labour and birth	0.60	2.79 (0.76)	51 (5.2)	253 (26.0)	522 (53.5)	149 (15.3)
7	I felt that I handled the situation well	0.66	2.78 (0.70)	42 (4.3)	246 (25.2)	575 (59.0)	112 (11.5)
21^c^	As a whole, how much control did you feel you had during childbirth?	0.55	2.57 (0.95)	167 (17.1)	236 (24.2)	419 (43.0)	153 (15.7)
	Cronbach’s alpha coefficient	0.78					
Domain: General support			3.09 (0.43)				
10	Both my partner and I were treated with warmth and respect	0.54	3.09 (0.57)	13 (1.3)	81 (8.3)	685 (70.3)	196 (20.1)
11	I received the information I needed during labour and birth	0.54	3.05 (0.50)	8 (0.8)	75 (7.7)	751 (77.0)	141 (14.5)
16	My impression of the team’s medical skills made me feel secure	0.54	3.12 (0.56)	9 (0.9)	74 (7.6)	685 (70.3)	207 (21.2)
	Cronbach’s alpha coefficient	0.70					
Domain: Perceived safety			2.69 (0.64)				
18^d^	I have many negative memories from childbirth	0.59	2.73 (0.70)	97 (10.0)	568 (58.3)	264 (27.1)	46 (4.7)
19^d^	Some of my memories from childbirth make me feel depressed	0.53	2.64 (0.73)	88 (9.0)	509 (52.2)	320 (32.8)	58 (6.0)
	Cronbach’s alpha coefficient	0.77					
Domain: Professional support			1.97 (0.54)				
12^d^	I would have preferred the midwife to be more present during labour and birth	0.23	2.02 (0.63)	9 (0.9)	179 (18.4)	613 (62.9)	174 (17.9)
13^d^	I would have preferred more encouragement from the midwife	0.29	1.91 (0.60)	7 (0.7)	114 (11.7)	635 (65.1)	219 (22.5)
	Cronbach’s alpha coefficient	0.72					
Total:			2.65 (0.34)				

Item 3, 5, 8, 9, 14, 15, 17, 20 and 22 were removed because of the result of the exploratory factor analysis

*SD* standard deviation

^a^For Cronbach’s alpha coefficients, corrected item-total correlations and the mean scores, all reversed items were recoded. Higher scores indicate a more positive attitude towards childbirth experience

^b^The response distribution of each individual item before the reserved item were recoded

^c^The items were assessed with visual analogue scales. The items were transformed into a 4-point Likert scale

^d^Items are reverse scored

### Known-group validity

The domains were compared among primiparous and multiparous participants and participants who received different intrapartum interventions (see Table 5). Primiparous mothers were less likely to receive *General support* (*d*=-0.16; *P*=.02), *Perceived safety* (*d*=-0.22; *P*=.002), *Professional support* (*d*=-0.24; *P*<.001) and the total scale score (*d*=-0.30; *P*<.001). Participants who received oxytocin augmentation were more likely to perceive safety and receive professional support. When comparing the participants who had spontaneous vaginal birth and operative birth, statistical differences were found in the domains *Own capacity* (*d*=0.19; *P*<.001) and *Professional support* (*d*=-0.16; *P*=.02). Participants, who had companionship during labour, had higher scores in all the domains and the total scale.

**Table 5:** Known-group comparison (*n*=975)

	Parity					
	Primiparous		Multiparous		Cohen’s D effect Size	*P* value
	n	Mean (SD)	n	Mean (SD)		
Own capacity	693	2.83 (0.50)	282	2.89 (0.51)	-0.12	.08
General support	693	3.07 (0.45)	282	3.14 (0.38)	-0.16	.02
Perceived safety	693	2.65 (0.66)	282	2.79 (0.59)	-0.22	.002
Professional support	693	1.93 (0.53)	282	2.06 (0.56)	-0.24	<.001
Total	693	2.62 (0.34)	282	2.72 (0.34)	-0.30	<.001
	Oxytocin augmentation					
	No		Yes		Cohen,s D effect Size	*P* value
	n	Mean (SD)	n	Mean (SD)		
Own capacity	408	2.83 (0.53)	567	2.86 (0.48)	-0.06	.33
General support	408	3.07 (0.44)	567	3.10 (0.43)	-0.06	.39
Perceived safety	408	2.79 (0.63)	567	2.62 (0.65)	0.26	<.001
Professional support	408	2.04 (0.55)	567	1.91 (0.53)	0.22	<.001
Total	408	2.68 (0.36)	567	2.62 (0.32)	0.17	.01
	Mode of birth					
	Spontaneous vaginal		Operative		Cohen,s D effect Size	*P* value
	n	Mean (SD)	n	Mean (SD)		
Own capacity	662	2.88 (0.47)	313	2.79 (0.56)	0.19	.01
General support	662	3.08 (0.46)	313	3.09 (0.46)	-0.03	.69
Perceived safety	662	2.68 (0.63)	313	2.70 (0.68)	-0.03	.71
Professional support	662	1.94 (0.55)	313	2.02 (0.54)	-0.16	.02
Total	662	2.65 (0.31)	313	2.65 (0.39)	-.02	.82
	Use of pain medication					
	No		Yes		Cohen,s D effect Size	*P* value
	n	Mean (SD)	n	Mean (SD)		
Own capacity	194	2.91 (0.55)	781	2.83 (0.49)	0.15	.07
General support	194	3.08 (0.41)	781	3.09 (0.44)	-0.03	.69
Perceived safety	194	2.68 (0.63)	781	2.69 (0.65)	-0.02	.84
Professional support	194	1.99 (0.54)	781	1.96 (0.55)	.06	.48
Total	194	2.66 (0.37)	781	2.64 (0.33)	0.06	.47
	Companionship during labour					
	No		Yes		Cohen,s D effect Size	*P* value
	n	Mean (SD)	n	Mean (SD)		
Own capacity	623	2.82 (0.51)	352	2.90 (0.48)	-0.17	.01
General support	623	3.02 (0.40)	352	3.20 (0.46)	-0.43	<.001
Perceived safety	623	2.57 (0.62)	352	2.90 (0.62)	-0.54	<.001
Professional support	623	1.91 (0.53)	352	2.06 (0.56)	-0.27	<.001
Total	623	2.58 (0.31)	352	2.77 (0.35)	-0.57	<.001

*SD* standard deviation

Higher scores indicate a more positive attitude towards childbirth experience

### Childbirth experiences in Chinese women

The mean of CEQ2.0-R was 2.65 (SD=0.34). Participants had received adequate general support. There were 91.5% of the participants agreed that their impression of the team’s medical skills made them feel secure. However, a high proportion of participants reported that they would prefer the midwives to be more present during labour and birth (80.8%) and having more encouragement from the midwives (87.6%).

## Discussion

This is the first study to examine the psychometric properties of the Chinese translated version of CEQ2.0. This study provides evidence for the reliability and validity of the translated CEQ2.0. The original model of CEQ2.0 showed a poor fit [11]. Therefore, a revised model CEQ2.0-R was constructed, which showed adequate validity and reliability. CEQ2.0-R is a self-report instrument, and it is easy to use. It took approximately five minutes to administer and can be used to evaluate childbirth experiences.

CEQ2.0-R measured the childbirth experiences in four domains (*Own capacity, General support, Perceived safety and Professional support*). Nine items were deleted from the original CEQ2.0. Not many variations were shown in the deleted items. Furthermore, three items were removed from the domain *Perceived safety* in CEQ2.0, and one item was shifted to another domain. The infant mortality rate was 1.4 per 1000 live birth, and the maternal mortality ratio was 0 in 2019 [23]. Therefore in Hong Kong, the infant mortality rate has been among the lowest in the world [24]. With this, participants in this population group may not perceive that safety or security in labour was a concern in Hong Kong. In addition, the domain *Participation* from the original model was removed, and one new domain *General support* was constructed in CEQ2.0-R. This domain measured the support from the general experiences during labour. It consists of the impression related to medical team skills, the support provided by the staff, and how the participants were treated in the hospital.

To our knowledge, CEQ2.0 was only validated in two Western countries, Sweden [11] and the United Kingdom [19]. Although the participants indicated that they had positive birthing experiences, the mean score was relatively low. The experiences were merely positive. Studies show that negative childbirth experiences are associated with adverse postnatal outcomes, such as postnatal depression [25] and post-traumatic stress symptoms [26]. Approximately 17% of postpartum women suffered from postpartum depression globally [27]. Therefore, it is important to understand how childbirth experiences would affect postpartum mental health outcomes. In addition, many participants indicated that they would like to receive more support from midwives. One study conducted in Hong Kong shows that women preferred nurses to meet their informational and individual needs, perform competent nursing skills, be approachable and have positive attitudes, and demonstrate cultural competence [28]. Effective interventions on improving childbirth experiences need to be developed.

Oxytocin augmentation and parity had small effects on the total childbirth experiences. However, having companions during labour can significantly improve childbirth experiences. A qualitative evidence synthesis shows that companionship is associated with positive childbirth experiences [29]. During the COVID-19 pandemic, childbirth companionship was suspended in public hospitals in Hong Kong [1]. A recent study conducted in Hong Kong shows that after the suspension of childbirth companionship, the proportion of women who received childbirth massages decreased while there was an increased prevalence in opioid pain medication use during the postpartum period [1]. In addition, a higher proportion of women developed depressive symptoms in the early postpartum period after the announcement of the coronavirus alert [1]. However, it is unclear whether the increase in the depressive symptoms was related to the change in hospital practices or the COVID-19 pandemic. Therefore, further studies are needed with the validated CEQ2.0-R is needed to deepen our understanding of the experience during the COVID-19 pandemic. This can also differentiate the effect of different government restrictions worldwide due to the COVID-19 pandemic on maternal health outcomes.

This study has several strengths and limitations. This study was the first study to assess the ability of CEQ2.0 in examining the childbirth experiences of Chinese women. The translated and validated CEQ2.0-R may be useful for measuring the childbirth experiences of Chinese women. Secondly, this study has a large sample size, which is sufficient for performing CFA [30]. However, there are also some limitations of this study. All questions were self-reported, instead of retrieved from the medical records. Data was collected based on participants’ recall of the use of intrapartum interventions, therefore recall bias may occur. Secondly, the psychometric properties of the Chinese version of CEQ2.0 are based on a convenience sample of Hong Kong mothers. The mothers who have positive childbirth experiences may be more in favor of participating in the study. In addition, our sample has a higher educational level and monthly family income when compared to the general population [31]. Therefore, the study results may not be generalizable to other populations within Hong Kong.

## Conclusions

CEQ2.0-R shows adequate psychometric performance in assessing women’s childbirth experiences. The questionnaire is easy to be administered and can be used to assess several domains of the childbirth experiences. This questionnaire may be useful for midwives to assess women's childbirth experiences in the postpartum period and evaluate the aspects of additional support needed during childbirth when providing health care to the general population.

### Availability of data and materials

The data analyzed in this study are not publicly available due to privacy policy, but are available from the corresponding author on reasonable request. For further information about data access, please contact the corresponding author, Kris Lok by email.

## Supplementary Information

**Additional file 1.**

## Data Availability

The data for this study is available on request to the corresponding author.

## Abbreviations

CEQ: Childbirth Experience Questionnaire
CEQ2.0: Childbirth Experience Questionnaire 2
CEQ2.0-R: Childbirth Experience Questionnaire 2 revised
CFI: Comparative Fit Index
CFA: Confirmatory Factor Analysis
EFA: Exploratory Factor Analysis
RMSEA: Root-mean-square Error of Approximation
SD: Standard Deviation
SRMR: Standardized root mean square residual
TLI: Tuker Lewis Index
KMO: Kaiser-Meyer-Olkin
VAS: Visual Analogue Scales
