# Supplementary figures and images for: Validating the use of the revised childbirth experience questionnaire in Hong Kong
Source: BMC Pregnancy Childbirth. 2022 Feb 15;22:126. doi: 10.1186/s12884-022-04456-x (PMC8845391; doi:10.1186/s12884-022-04456-x)

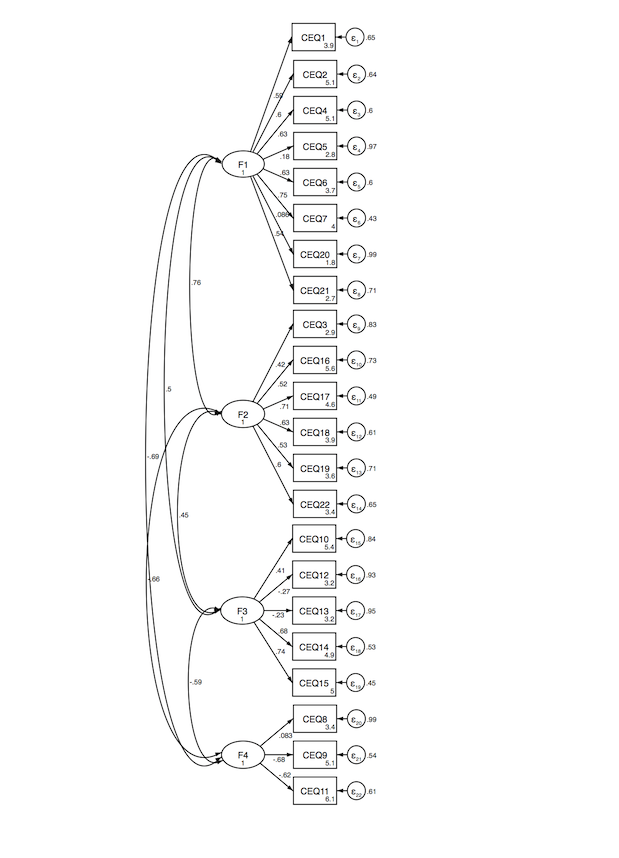

Supplement: Supplementary file 1 — Additional file 1. [file 12884_2022_4456_MOESM1_ESM.png]
